# Lipoproteins Cause Bone Resorption in a Mouse Model of *Staphylococcus aureus* Septic Arthritis

**DOI:** 10.3389/fmicb.2022.843799

**Published:** 2022-03-09

**Authors:** Michelle Schultz, Majd Mohammad, Minh-Thu Nguyen, Zhicheng Hu, Anders Jarneborn, Carina M. Wienken, Matti Froning, Rille Pullerits, Abukar Ali, Heiko Hayen, Friedrich Götz, Tao Jin

**Affiliations:** ^1^Department of Rheumatology and Inflammation Research, Institute of Medicine, Sahlgrenska Academy, University of Gothenburg, Gothenburg, Sweden; ^2^Institute of Medical Microbiology, University Hospital of Münster, Münster, Germany; ^3^Department of Microbiology and Immunology, The Affiliated Hospital of Guizhou Medical University, Guiyang, China; ^4^Department of Rheumatology, Sahlgrenska University Hospital, Gothenburg, Sweden; ^5^Institute of Inorganic and Analytical Chemistry, University of Münster, Münster, Germany; ^6^Department of Clinical Immunology and Transfusion Medicine, Sahlgrenska University Hospital, Gothenburg, Sweden; ^7^Department of Microbial Genetics, Interfaculty Institute of Microbiology and Infection Medicine Tübingen (IMIT), University of Tübingen, Tübingen, Germany

**Keywords:** *Staphylococcus aureus*, lipoproteins, lipopeptides, septic arthritis, bone mineral density, mouse

## Abstract

Septic arthritis, most often caused by *Staphylococcus aureus*, is a rapidly progressive and destructive joint disease with substantial mortality and morbidity. *Staphylococcus aureus* lipoproteins (Lpps) are known to induce arthritis and bone destruction. Here, we aimed to investigate the bone resorptive effect of *S. aureus* Lpps in a murine arthritis model by intra-articular injection of purified *S. aureus* Lpps, synthetic lipopeptides, and live *S. aureus* strains. Analyses of the bone mineral density (BMD) of the distal femur bone were performed. Intra-articular injection of both live *S. aureus* and purified *S. aureus* Lpps were shown to significantly decrease total- and trabecular BMD. Liquid chromatography–mass spectrometry analyses revealed that the Lpps expressed by *S. aureus* SA113 strain contain both diacyl and triacyl lipid moieties. Interestingly, synthetic diacylated lipopeptide, Pam_2_CSK_4_, was more potent in inducing bone resorption than synthetic triacylated lipopeptide, Pam_3_CSK_4_. Modified lipoproteins lacking the lipid moiety were deprived of their bone resorptive abilities. Monocyte depletion by clodronate liposomes fully abrogated the bone resorptive capacity of *S. aureus* lipoproteins. Our data suggest that *S. aureus* Lpps induce bone resorption in locally-induced murine arthritis, an effect mediated by their lipid-moiety through monocytes/macrophages.

## Introduction

Septic arthritis remains one of the most dangerous joint diseases due to its rapidly progressive and destructive nature (Sharff et al., 2013). The most common pathogen associated with bacterial arthritis is *Staphylococcus aureus* (Kaandorp et al., 1997). Despite advances in the use of antibiotics, up to 50% of patients experience permanent joint dysfunction (Jin et al., 2021). Septic arthritis might be associated with certain comorbidities such as osteoporosis, as in a mouse model septic arthritis caused by intravenous (i.v.) injection of *S. aureus* leads to a rapid systemic bone resorption (Verdrengh et al., 2006).

Increasing evidence has demonstrated bacterial components cause joint inflammation and bone destruction. Antibiotic-killed *S. aureus* is known to induce synovitis and destructive arthritis, which is triggered by the bacterial cell wall debris (Ali et al., 2015c). The lipoproteins (Lpps) contained in the cell wall of *S. aureus* consist of a protein- and a lipid-moiety (Nguyen et al., 2020). The activation of pattern recognition receptors, such as Toll-like receptors (TLRs), is stimulated by the lipid-moiety of *S. aureus* Lpps which consequently initiates a strong innate immune system response (Nguyen and Gotz, 2016). Lately, *S. aureus* Lpp has been shown to play an important role in various infectious or inflammatory *in vivo* settings (Mohammad et al., 2019, 2020, 2021; Kopparapu et al., 2021). In a local murine knee joint model (Mohammad et al., 2019), the initiated innate immune response has a profound impact on the inflammation and destruction of joints. This effect is primarily mediated by the recruitment of monocytes/macrophages (Mohammad et al., 2019), which are the cells that can differentiate into osteoclasts and induce bone resorption (Boyle et al., 2003). An imbalance in the bone remodeling process, caused by an excess of osteoclasts and a deficit of osteoblasts, can induce osteoporosis (Fischer and Haffner-Luntzer, 2021). Currently the golden standard used to determine osteoporosis is measurement of bone mineral density (BMD). Trabecular composition is known to determine the strength of bone and consequently impacts several skeletal conditions (Adams, 1992). Osteoclasts and osteoblasts which are responsible for maintaining bone homeostasis, express a variety of TLRs. Bacterial Lpps, ligands to TLRs, can therefore affect the differentiation and activation of osteoclasts and osteoblasts. *Staphylococcus aureus* produces both diacylated and triacylated Lpps depending on the environment (Kurokawa et al., 2009, 2012). Diacylated and triacylated Lpps induce signal transduction through the TLR2/TLR6 and TLR2/TLR1 heterodimers, respectively (Jin et al., 2007; Kang et al., 2009). The synthetic lipopeptides, Pam_2_CSK_4_ (Pam2Cys) and Pam_3_CSK_4_ (Pam3Cys), imitate the lipid-moiety portion of diacylated and triacylated bacterial Lpps, respectively, and can therefore induce the same signaling response as original structures of Lpps (Kang et al., 2009). We hypothesize that *S. aureus* Lpp is one of the driving forces for bone resorption in septic arthritis.

In the present study, we used the murine septic arthritis model to investigate bone resorption induced by *S. aureus* Lpps in septic arthritis. In accordance with our hypothesis, both the live *S. aureus* LS-1 strain and the purified *S. aureus* Lpps, induced BMD loss, in a lipid-moiety dependent manner. We found that the Lpps expressed by *S. aureus* SA113 strain contain both diacyl and triacyl lipid moieties. The diacylated lipid-moiety of the synthetic lipopeptide Pam2Cys was more potent in inducing bone resorption than the triacylated lipid-moiety, Pam3Cys. Finally, monocytes, and not neutrophils, were determined to be the culprits behind *S. aureus* Lpp-induced bone loss.

## Materials and Methods

### Mice

Eight- to 12-week-old female NMRI mice were purchased from Envigo (Venray, Netherlands) and stored under standard temperature, light, and nutrition conditions in the animal facility of the Department of Rheumatology and Inflammation Research, University of Gothenburg.

### Preparation of Bacterial Solutions

The *S. aureus* LS-1 (Bremell et al., 1991) and *S. aureus* Newman strains were prepared as described. The bacterial strains were stored at a temperature of −70°C. Upon use, the solution was thawed, washed with sterile phosphate-buffered saline (PBS), and adjusted to the concentration of 1 × 10^4^ colony-forming units (CFU)/knee for the LS-1 strain experiments or into a concentration of 4 × 10^3^ CFU/knee for the Newman strain experiments.

### Expression and Purification of Lpl1(+sp) and Lpl1(−sp)

*Staphylococcus aureus* lipoproteins Lpl1(+sp) and Lpl1(−sp) were prepared and purified, as previously described (Nguyen et al., 2015). Lpl1(+sp) and Lpl(−sp) were extracted from the membrane fraction of *S. aureus* SA113 (pTX30::lpl1-his) and cytoplasmic fraction of *S. aureus* SA113Δ*lgt* (pTX30::lpl1(−sp)-his) respectively, as previously explained (Mohammad et al., 2019). Furthermore, SDS-PAGE was used to confirm the Lpl1-his purification, as conducted previously (Nguyen et al., 2015). A freezer was prepared to a temperature of −70°C to store the purified compounds of Lpl1(+sp) and Lpl1(−sp) until usage. Subsequently, before each experiment the compounds were diluted in sterile PBS to a concentration of 10 μg/knee that is known to be able to induce synovitis in mice if injected intraarticularly (Mohammad et al., 2019).

### Experimental Protocols for the Induction of Arthritis With Staphylococcal Lipoproteins

To study the impact of *S. aureus* Lpp on murine BMD, six sets of experiments were performed. One of the following materials was prepared in 20 μl of PBS and intra-articularly (i.a.) injected into the knee joints of NMRI mice: (1) live *S. aureus* LS-1 strain or PBS; (2) live *S. aureus* Newman strain or PBS; (3) purified Lpl1(+sp) or Lpl1(−sp) *S. aureus* Lpps, or PBS; (4) synthetic lipopeptides, Pam_2_CSK_4_ and Pam_3_CSK_4_ (EMC, Tübingen, Germany), or PBS; (5) purified Lpl1(+sp) or PBS in monocyte/macrophage- and neutrophil depleted mice; or (6) purified Lpl1(+sp) in mice treated with TNF-α inhibitor, IL-1 receptor antagonist, or PBS as a treatment control group. The diameter of the knee joints of mice was measured regularly with a caliper to determine the severity of the induced arthritis.

### *In vivo* Cell Depletion Procedures

The selective elimination of macrophages can be obtained through the use of clodronate liposomes (Liposoma BV, Netherlands; Van Rooijen and Sanders, 1994). To deplete both synovial residual macrophages as well as systemic monocytes, NMRI mice were i.a. injected with 20 μl of clodronate liposomes into the knee joints followed by intravenous injection with 200 μl of clodronate liposomes (Liposoma BV, Netherlands), respectively. The i.a. injection for the local depletion was performed 1 day prior to exposing the knee joints with either Lpl1(+sp) or PBS, while the intravenous injections for the systemic depletion were performed on the day before the experiment, and continued onto days 1, 3, and 5 of the experiment after exposure to either Lpl1(+sp) or PBS. In the same manner, PBS control liposomes (Liposoma BV, Netherlands) were injected into another set of mice in order to serve as controls, as previously described (Mohammad et al., 2019).

For the selective depletion of murine blood neutrophils, the monoclonal antibody Anti-Ly6G (clone 1A8; BioXCell) was used (Daley et al., 2008). A dose of 400 μg of anti-Ly6G or isotype control (clone 2A3; BioXCell) in 200 μl of PBS/mouse was injected intraperitoneally into NMRI mice to deplete the mice of systemic neutrophils, or to serve as controls, respectively. The injections were performed 1 day prior to exposing the knee joints with either Lpl1(+sp) or PBS, and continued onto days 1 and 4 after exposure to either Lpl1(+sp) or PBS, as previously explained (Mohammad et al., 2019).

### Treatment With TNF-α Inhibitor or IL-1 Receptor Antagonist in NMRI Mice in *S. aureus* Lpp-Induced Bone Loss

The soluble TNF receptor, Etanercept (Enbrel; Wyeth Europa), which is known to fully inhibit the biological function of murine TNF (Fei et al., 2011; Ali et al., 2015b), was used in the experiment as anti-TNF treatment. Subcutaneous (s.c.) injections of Etanercept (0.2 mg/mouse in 0.1 ml of PBS) were performed. The administration began 2 days prior to exposing the knee joints with Lpl1(+sp), and continued on the day the knee joints where i.a. injected with Lpl1(+sp), and were followed by every other day until the termination date. PBS was s.c. injected as control treatment on days when Etanercept was not administered.

To block the function of IL-1 in the murine model, the IL-1 receptor antagonist Anakinra (Kineret; Amgen) was used as anti-IL-1 treatment (Sgroi et al., 2011; Ali et al., 2015a). Anakinra (1 mg/mouse in 0.1 ml of PBS) was s.c. administered daily, starting 2 days prior to exposing the knee joints with Lpl1(+sp), and continued until the termination date.

Phosphate-buffered saline was administered s.c. daily in the control treatment group, starting 2 days prior to exposing the knee joints with Lpl1(+sp), and continued until termination day.

### Microcomputed Tomography

Following the termination date of the experiments, the mice knee joints were suspended in 4% formaldehyde for at least 7 days. To detect induced bone loss, mice knee joints were transferred to PBS and subsequently scanned *ex vivo* in the Skyscan1176 microcomputed tomography (micro-CT) scanner (Bruker, Antwerp, Belgium). During scanning, the voltage and current were set to 55 kV and 455 μA, respectively, with a voxel size of 9 μm and an aluminum filter of 0.2 mm and an exposure time of 755 ms. The scanning angular rotation was set to 180°, and the X-ray projections were retrieved at 0.42° intervals.

To obtain the BMD of mice knee joints, a morphometric analysis was performed on the distal femur bone. A 0.86 mm section of the distal femur was obtained from 100 micro-CT image slices, 50 slices above and below the reference point, for this experiment the growth plate was selected to be the reference point. The morphometric analysis was performed with the CT-Analyzer as previously described (Fatima et al., 2017).

### Detection and Structure Elucidation of Lpl1(+sp) by High Resolution-Mass Spectrometry

For the tryptic digestion of the Lpl1(+sp), sequencing grade trypsin modified from porcine pancreas was obtained from SERVA Electrophoresis GmbH (Heidelberg, Germany). In total, 50 μl of the purified Lpl1(+sp) solution (1 mg/ml) was denatured by heating at 95°C for 5 min. Around 50 μl trypsin (0.02 μg/μl in 50 mM NH_4_HCO_3_, 10% ACN) and 50 μl of 50 mM NH_4_HCO_3_ were added, and the protein was digested at 37°C for 18 h. The digested protein was dried using a SpeedVac (Thermo Fisher, Waltham, MA, United States) and reconstituted in 100 μl MeOH/H_2_O 1:1 (v/v).

For chromatographic separation, a 1,100 series from Agilent Technologies (Santa Clara, CA, United States) was employed. The system consists of an autosampler (G1329A), a binary pump (G1312A), a degasser (G1316A), and a column compartment (G1316A). Instrument control was established by ChemStation. For sample analysis, an Xselect CSH C18 (100 × 2.1, 3.5 μm, 130 Å) from Waters™ Corporation (Milford, MA, United States) was used at 40°C with a flow rate of 0.3 ml/min. Mobile phase A consisted of 0.05% aqueous TFA and mobile phase B was composed of 0.05% TFA in ACN/IPA 1:1. The separation started at 25% B for 0.5 min, followed by an increase to 95% B in 6.5 min. To elute all the analytes and clean the column, 95% B was maintained for 20 min. After a steep decrease to 25% B in 0.5 min, the column was re-equilibrated for 7.5 min. The injection volume was 2 μl.

For high resolution mass spectrometric detection and MS/MS experiments, a Q Exactive™ Plus Hybrid Quadrupole-Orbitrap™ (Thermo Fisher Scientific, Waltham, MA, United States) mass spectrometer was operated in positive and negative electrospray ionization mode, respectively. A resolving power of 70,000 (FWHM @ *m*/*z* 200) was used to analyze the mass range *m*/*z* 113.4–1700.0. For data-dependent MS/MS experiments, the three most intense ions were fragmented with collision energy of 30 eV normalized at *m*/*z* 500. The instrument was controlled using the software Xcalibur™ 4.1 (Thermo Fisher Scientific™, Waltham, MA, United States). Unless stated otherwise in the figure description, the Q Exactive Plus was used for detection.

The data analysis of the high-resolution measurements was facilitated by the open-source software mzMine 2 (version 2.53; Pluskal et al., 2010). The accurate masses were detected with a noise level of 10,000 using the exact mass algorithm. For chromatogram building, the ADAP chromatogram builder was used (Myers et al., 2017). The minimum group size in number of scans was set to 4, the group intensity threshold to 50,000 and the minimum highest intensity to 10,000. The *m/z* tolerance was set to 0.001 *m/z* or to 15 ppm, whichever is higher. Savitzky–Golay smoothing with a filter width of 5 was applied, followed by chromatogram deconvolution using the wavelets (ADAP) algorithm. For deconvolution, the S/N was estimated using the intensity window and the S/N set to 3. The minimum feature height was set to 10,000 and the coefficient/area threshold set to 100. The retention time wavelet range was set from 0.00 to 0.15, and the median m/z center was calculated. Subsequently, isotopic peaks were grouped within an m/z tolerance of 0.001 m/z and 5 ppm, whichever is higher. The retention time tolerance was set to 0.1 min and the isotope with the lowest *m/z* was saved.

### Statistical Analysis

All statistical analyses were performed using GraphPad Prism version 8.4.2 software for Macintosh (GraphPad Software, La Jolla, CA, United States). To compare the outcome between different treatment groups and different animals the Mann–Whitney *U* test was used, while the two-tailed paired *t* test was used by comparing the contralateral knee joints within the same animal to determine the statistical significance of the collected data. All data are expressed as mean ± SEM. *p < 0.05, **p < 0.01, ***p < 0.001, and ****p < 0.0001.

## Results

### Intra-Articular Injection of *Staphylococcus aureus* LS-1 Strain Induces Bone Resorption

**Figure 1:**
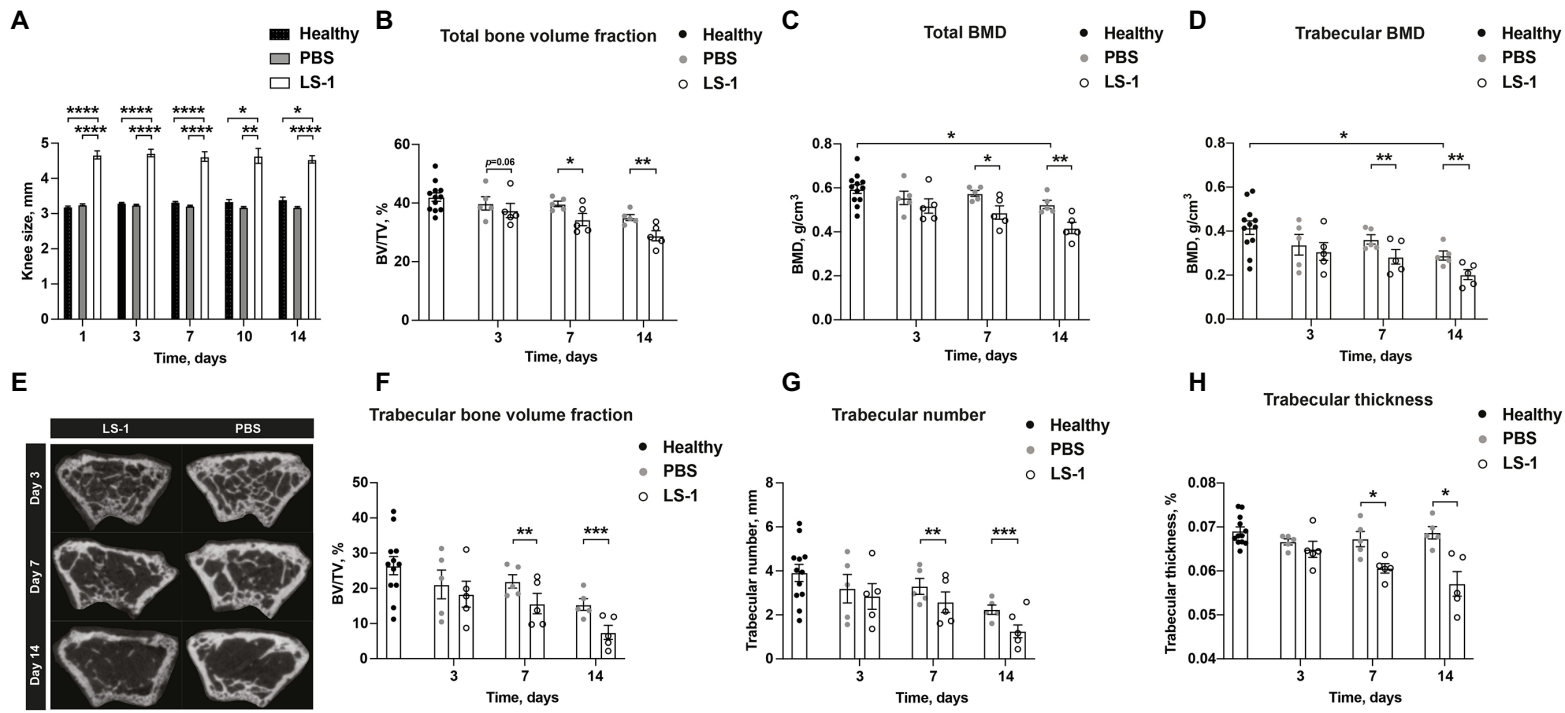
Intra-articular injection of *Staphylococcus aureus* LS-1 strain induces bone resorption. **(A)** Measurement of knee swelling (mm) in NMRI mice up to 14 days after intra-articular (i.a.) injection with 20 μl of *Staphylococcus aureus* (*S. aureus*) LS-1 strain (1 × 10^4^ CFU/knee) or 20 μl of phosphate-buffered saline (PBS; *n* = 5/group), compared with healthy NMRI mice (*n* = 6; *n* = 12 knee joints) i.a. injected with 20 μl of PBS. **(B)** The total bone volume fraction [bone volume/tissue volume (BV/TV)] measured in NMRI mice up to 14 days after i.a. injection with 20 μl of *S. aureus* LS-1 strain [1 × 10^4^ CFU/knee] or 20 μl of PBS (*n* = 5/group), compared with healthy NMRI mice (*n* = 6; *n* = 12 knee joints) i.a. injected with 20 μl of PBS. **(C)** The total- and **(D)** trabecular bone mineral density (BMD, g/cm^3^). **(E)** Representative 2D microcomputed tomography (micro-CT) images of the femoral metaphysis of mice i.a. injected with *S. aureus* LS-1 (left panel) and PBS (right panel) on days 3 (upper), 7 (middle), and 14 (lower) post injection. Graphs showing quantitative evaluation of **(F)** trabecular bone volume fraction (BV/TV), **(G)** trabecular number, and **(H)** trabecular thickness. Statistical evaluations were performed using the Mann–Whitney *U* test between healthy control mice and PBS injected *S. aureus* infected mice **(A–D,F–H)** or Paired *t* test between *S. aureus* and PBS injected mice originating from the same host **(A–D,F–H)**. The data are presented as a bar **(A)** or scatterplot with data expressed as the mean ± SEM (**B–D**,**F–H**; ^*^p < 0.05, ^**^p < 0.01, ^***^p < 0.001, and ^****^p < 0.0001).

To study the effects of *S. aureus* on BMD in mice with septic arthritis, NMRI mice were i.a. injected with either *S. aureus* LS-1 strain, a pathogenic *S. aureus* strain that was originally isolated from a mouse that spontaneously developed septic arthritis (Bremell et al., 1991), or with PBS, and control NMRI mice were injected with PBS in both knees. Subsequently, a micro-CT evaluation was performed on the femoral BMD of mice with *S. aureus* septic arthritis. As expected, swelling in the joints infected with *S. aureus* started already on day 1 and continued until day 14 (Figure 1A). Importantly, we observed that the i.a. injected *S. aureus* LS-1 strain significantly reduced total- and trabecular femoral BMD in mice when compared to contralateral knees injected with PBS, from 7 to 14 days post infection (Figures 1C,D). A trend toward BMD reduction, although not statistically significant was observed already on day 3 post infection (Figures 1C,D). These results imply that *S. aureus*-induced septic arthritis causes bone resorption in murine knee joints. Additionally, significant differences in total- and trabecular BMD were observed between the healthy mouse femoral bone and the femoral bone from PBS injected knee joints from the mice infected with *S. aureus* in their collateral knees (Figures 1C,D), suggesting that systemic inflammation caused by *S. aureus* local infection has significant impact on the bone metabolism. Representative imagines of the decrease in BMD, verified by micro-CT evaluation, were obtained on days 7 and 14 after i.a. injection with *S. aureus* (Figure 1E). Similarly, *S. aureus* LS-1 i.a. injection significantly reduced total- and trabecular bone volume fraction (bone volume/tissue volume, Figures 1B,F), trabecular number (Figure 1G), and trabecular thickness (Figure 1H) on day 7 as well as day 14 post infection. At the early stage of the infection (on day 3), total- and trabecular bone volume fraction, trabecular number, as well as trabecular thickness were not significantly affected (Figures 1B,F–H). To assure our finding is not LS-1 strain-specific, another widely-used laboratory *S. aureus* Newman strain was injected to the mouse knee joints and BMD was analyzed on day 10 post infection. Both total and trabecular BMD were significantly downregulated in *S. aureus* Newman infected joints compared to healthy joints, suggesting that our finding is universal for different *S. aureus* strains (Supplementary Figure 1).

### Intra-Articular Injection of *Staphylococcus aureus* Lpp Induces Bone Mineral Density Loss in a Lipid-Moiety Dependent Manner

**Figure 2:**
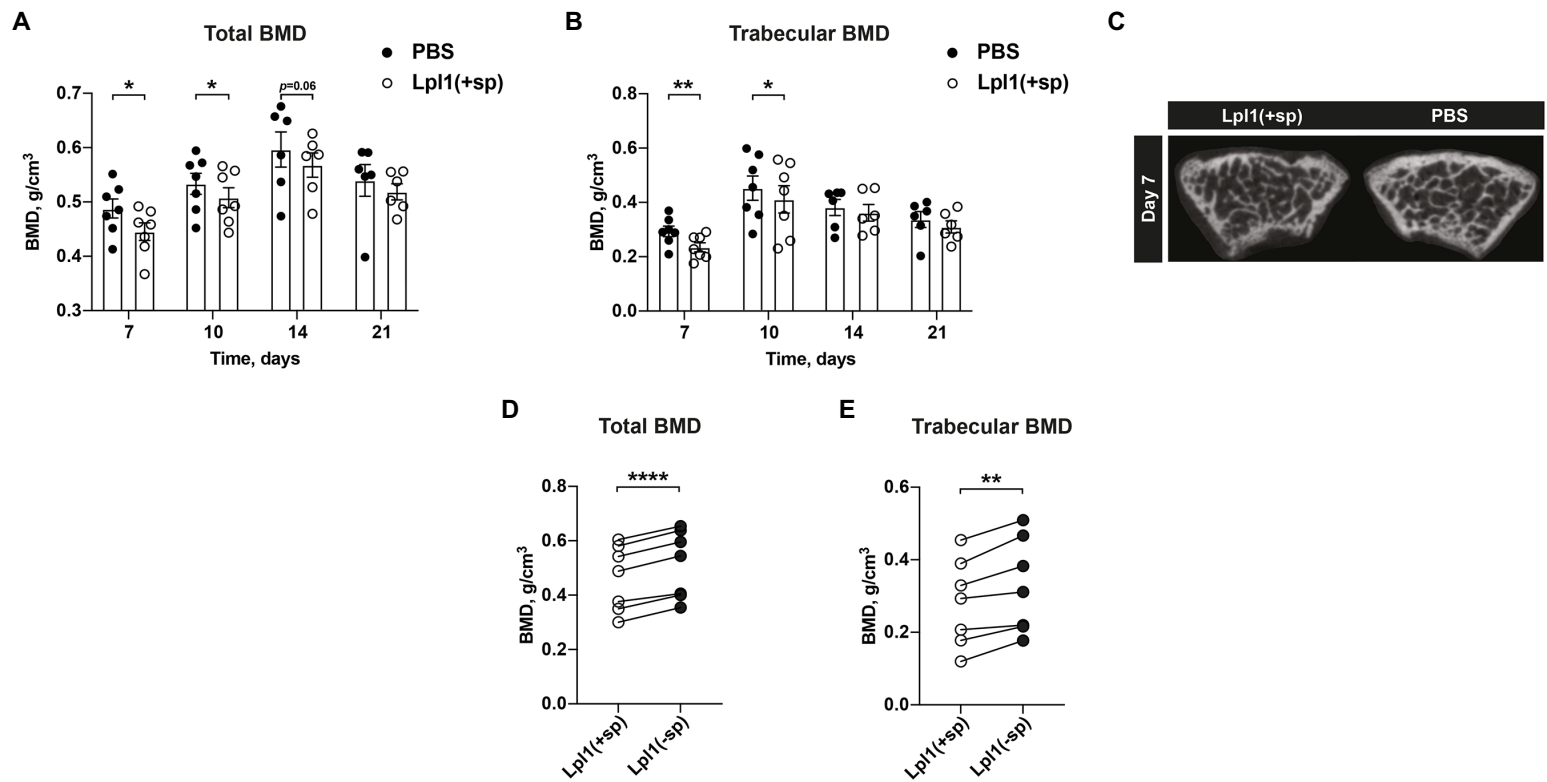
Intra-articular injection of *Staphylococcus aureus* Lpp induces BMD loss in a lipid-moiety dependent manner. **(A)** The total- and **(B)** trabecular BMD (g/cm^3^) measured on day 7, 10, 14, and 21 post intra-articular (i.a.) knee joint injection of 20 μl of purified *S. aureus* lipoprotein Lpl1(+sp; 10 μg/knee) or 20 μl of PBS, in NMRI mice (*n* = 6–7/group). **(C)** Representative micro-CT images of the mice knee joints i.a injected with *S. aureus* Lpl1(+sp; left panel) or PBS (right panel) on day 7. Measurements of the **(D)** total- and **(E)** trabecular BMD on day 7 of mice knee joints (*n* = 7/group) i.a. injected with 20 μl of intact purified *S. aureus* lipoproteins Lpl1(+sp; 10 μg/knee), or with 20 μl of lipoproteins lacking the lipid-moiety Lpl1(−sp; 10 μg/knee). The data were pooled from two independent experiments. Statistical evaluations were performed using the Paired *t* test **(A,B,D,E)**, with data expressed as the mean ± SEM (^*^p < 0.05, ^**^p < 0.01, and ^****^p < 0.0001).

*Staphylococcus aureus* Lpp is known to be one of the most potent bacterial components causing joint inflammation and bone destructions in septic arthritis (Mohammad et al., 2019). To further study whether the *S. aureus* Lpp induces loss of BMD, mice knee joints were i.a. injected with intact purified Lpl1(+sp) or PBS. Lpl1(+sp) is a model Lpp carrying the lipid moiety, which is derived from the νSaα-specific lipoprotein-like cluster (*lpl*) that exists in highly pathogenic and epidemic *S. aureus* strains (Nguyen et al., 2015). Significantly lower total- and trabecular BMD was observed in knees injected with Lpp compared to PBS on day 7 and 10 (Figures 2A,B). On the later time point (day 14 and 21) when the joint inflammation caused by Lpp was less pronounced, the difference of BMD in Lpp and PBS injected knees disappeared (Figures 2A,B). Representative images of the BMD loss caused by the *S. aureus* Lpp were obtained on day 7 post Lpp injection (Figure 2C). To elucidate the molecular mechanism of BMD loss caused by the *S. aureus* Lpp, mice knee joints were i.a. injected with intact Lpl1(+sp) carrying the lipid moiety, or as control with Lpl1(−sp) which lacks the lipid-moiety. An analysis of the total- and trabecular BMD in femoral bone was subsequently performed 7 days after Lpp injection (Figures 2D,E). The mice injected with intact *S. aureus* Lpl1(+sp) displayed a significantly lower total- and trabecular BMD in comparison to the Lpl1(−sp) injected ones (Figures 2D,E). These results demonstrate that the lipid moiety in *S. aureus* Lpp is absolutely necessary for induction of bone loss.

### Lpl1(+sp) Isolated From SA113 Contain Both Diacyl and Triacyl Lipid Moieties

To identify the lipid moiety structure, the tryptic Lpl1(+sp) was subjected to LC–MS analysis. As a result, two different tryptic lipopeptides can be detected; one cut directly after the first lysine (lipid-CGK), the other was cut after the second lysine (lipid-CGKGNETK) of the amino acid sequence of Lpl1. The detected short lipopeptides are mostly singly charged, the more abundant CGKGNETK lipopeptides can be detected as singly and doubly protonated molecules, with the doubly charged ones being more intensive. Both short and long peptides contain a mixture of lipid moieties with two-acyl and three-acyl residues, the latter carries in addition to the two diglyceride fatty acids also the *N-*acyl fatty acid. Supplementary Table 1 lists the lipopeptides identified using high resolution-MS. Assignments are based on chromatographic behavior and accurate mass (relative mass deviation less than 5 ppm compared to calculated theoretical mass). In addition, MS/MS experiments were performed for confirmation, using peptide fragments and neutral losses of fatty acids can be used to identify the lipopeptide structures.

### The Diacylated Lipid-Moiety of the Synthetic Lipopeptide, Pam2Cys, Is More Potent in Inducing Bone Resorption Than the Triacylated Lipid-Moiety, Pam3Cys

**Figure 3:**
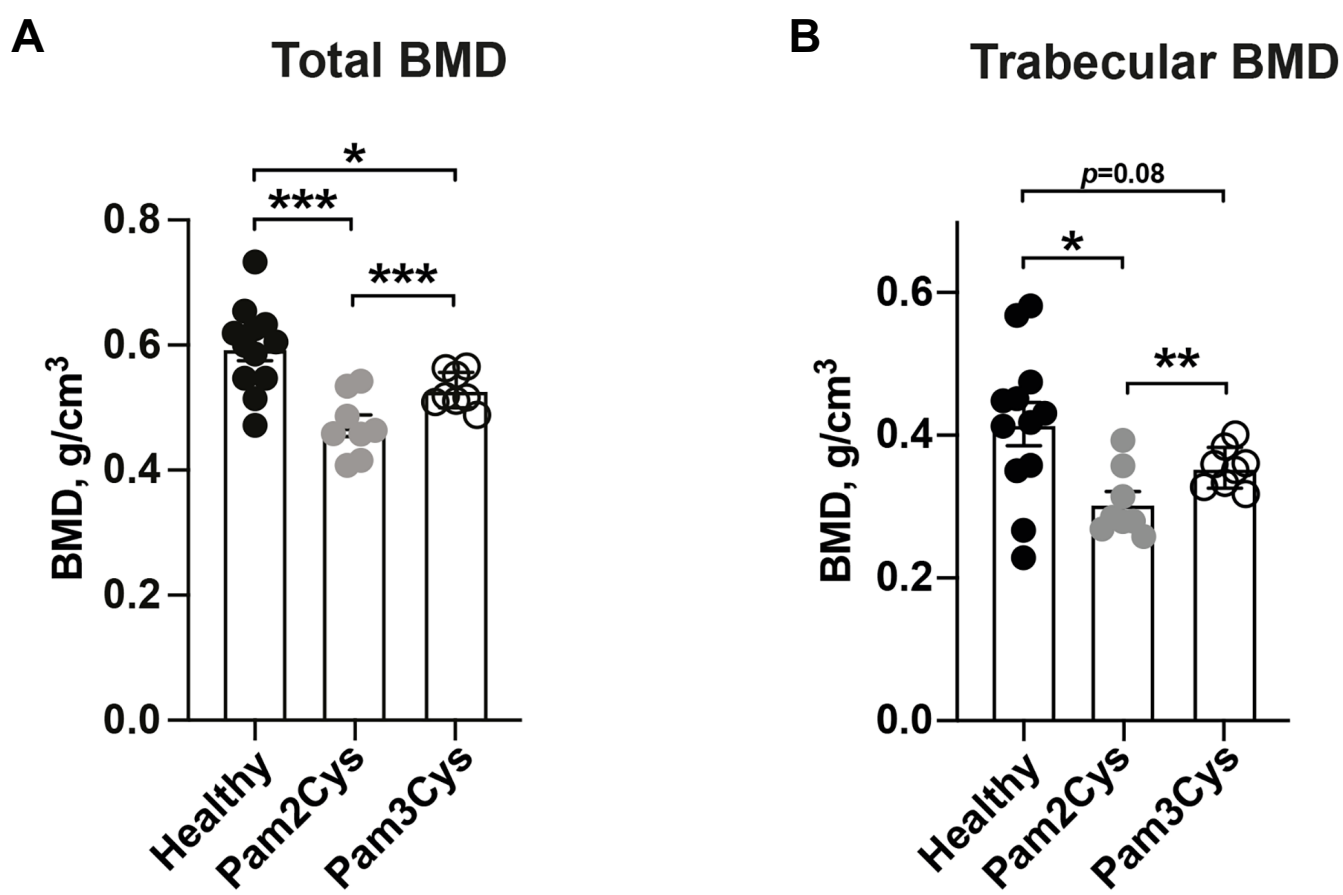
The diacylated lipid-moiety of the synthetic lipopeptide, Pam2Cys, is more potent in inducing bone resorption than the triacylated lipid-moiety, Pam3Cys. Graphs showing quantitative evaluations of **(A)** total- and **(B)** trabecular BMD (g/cm^3^) of femoral mice bone (*n* = 8–12/group) on day 7 post i.a. knee injection with either Pam3Cys or Pam2Cys, or PBS collaterally in healthy control mice. Data were pooled from four independent experiments. Statistical evaluations were performed using the Paired *t* test between Pam2Cys and Pam3Cys injected knee joints **(A,B)** or Mann–Whitney *U* test between healthy control mice and Pam2Cys or Pam3Cys injected knee joints **(A,B)**, with data expressed as the mean ± SEM (^*^p < 0.05, ^**^p < 0.01, and ^***^p < 0.001).

Since Lpl1(+sp) contained a mixture of diacyl and triacyl lipid moieties, we further compared the bone degenerative capabilities of the synthetic Pam2Cys and Pam3Cys lipopeptides that mimic the bacterial diacylated and triacylated Lpps respectively, on murine BMD. Mice knee joints were i.a. injected with 2 μg of either Pam2Cys or Pam3Cys. On day 7, we observed that Pam2Cys reduced the total- and trabecular BMD in mice significantly more than Pam3Cys (Figures 3A,B). As expected, the total BMD in femoral bone was significantly reduced by both i.a. injected Pam2Cys and Pam3Cys when compared to PBS i.a injected knees from healthy mice (Figure 3A). Regarding the trabecular BMD, Pam3Cys tended to reduce the BMD (*p* = 0.08), whereas Pam2Cys caused a significant reduction when compared to the knee joints of control mice injected with PBS (Figure 3B).

### Monocytes, and Not Neutrophils, Are the Culprits Behind *S. aureus* Lpp-Induced Bone Destruction

**Figure 4:**
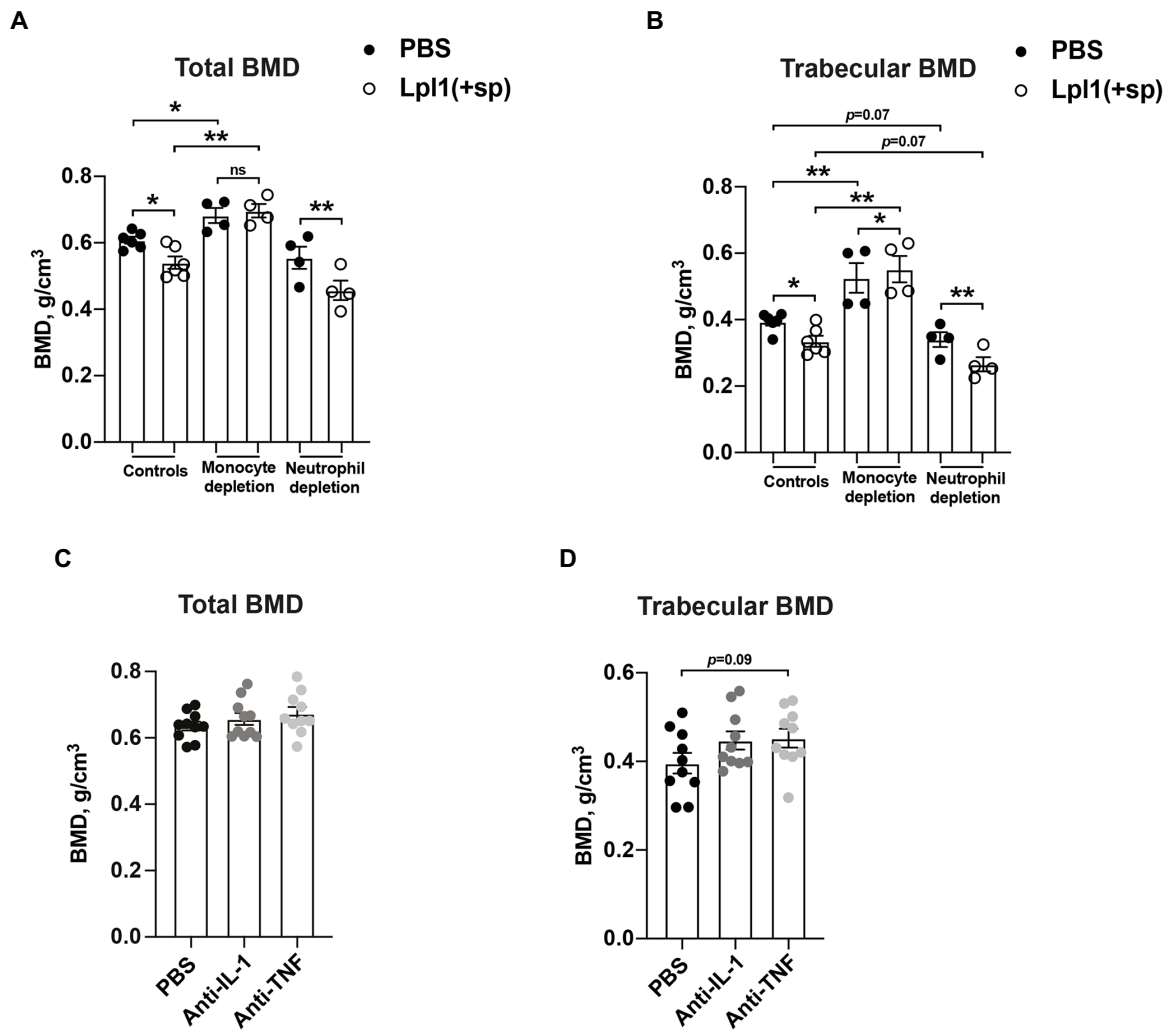
Monocytes, and not neutrophiles, are the culprits behind *Staphylococcus aureus* Lpp-induced bone destruction. **(A)** Total- and **(B)** trabecular BMD (g/cm^3^) of femoral mouse knee joints 7 days post intra-articular (i.a.) injection with 20 μl of purified *S. aureus* lipoprotein Lpl1(+sp; 10 μg/knee) or phosphate-buffered saline (PBS), measured in NMRI control mice (*n* = 6 knee joints/group) or NMRI mice depleted of either monocytes/macrophages using clodronate liposomes (*n* = 4 knee joints/group) or neutrophils using anti-Ly6G antibodies (*n* = 4 knee joints/group). **(C)** Total- and **(D)** trabecular BMD of femoral mouse knee joints 10 days post i.a. injection with 20 μl of purified Lpl1(+sp; 10 μg/knee), measured in NMRI mice treated with either anakinra, an interleukin 1 receptor antagonist (anti-IL-1) or etanercept, and a tumor necrosis factor inhibitor (anti-TNF) or PBS as control (*n* = 10 knee joints/group). Statistical evaluations were performed using the Paired *t* test between PBS and Lpl1(+sp) injected mice originating from the same host **(A,B)** or Mann–Whitney *U* test between PBS and Lpl1(+sp) injected mice of different treatment groups **(A,B)** or between PBS, anti-IL-1, or anti-TNF injected mice **(C,D)**, with the data presented as a scatterplot with data expressed as the mean ± standard error of the mean (^*^p < 0.05 and ^**^p < 0.01, ns = not significant).

Monocytes/macrophages, but not neutrophils, are known to be the immune cells responsible for the onset of *S. aureus* Lpp-induced arthritis (Mohammad et al., 2019). To further study the effect of monocyte/macrophage or neutrophil depletion on bone loss caused by Lpp, control mice or mice depleted of monocytes/macrophages by clodronate liposomes, or depleted of neutrophils by Ly6G antibodies, were i.a. injected into the knee with 10 μg of purified *S. aureus* Lpl1(+sp) or PBS. Injection of Lpl1(+sp) into knees in both control mice and neutrophil depleted mice caused a significantly lower total- and trabecular BMD in femoral bone than PBS (Figures 4A,B).

Intriguingly, the depletion of monocytes/macrophages by clodronate liposomes gave rise to no significant differences in total BMD between Lpl1(+sp) and PBS i.a. injected knee joints 7 days post injection (Figure 4A). However, a significant difference in trabecular BMD was observed between Lpl1(+sp) and PBS i.a. injected knee joints 7 days post injection (Figure 4B). Our data suggest that monocytes/macrophages might be the main culprits causing BMD loss in Lpp injected knees. Significantly lower total- and trabecular BMD between Lpl1(+sp) of control mice compared to monocyte/macrophage depleted mice, and PBS of control mice compared to monocyte/macrophage depleted mice, was observed on day 7 (Figures 4A,B), demonstrating the efficacy of clodronate in preventing bone absorption. Interestingly, total- and trabecular BMD tended to be lower in both Lpl1(+sp) and PBS injected knees from neutrophil depleted mice compared to control mice, indicating that neutrophils might be somehow bone protective (Figures 4A,B).

Monocytes and macrophages are one of the main producers of TNF-α and IL-1. Both cytokines are implicated in the development of osteoporosis (Kwan Tat et al., 2004). To further study the effects of IL-1 and TNF on bone absorption in Lpp-induced arthritis, *S. aureus* Lpp i.a. injected mice were treated with IL-1 or TNF inhibitors and BMD was subsequently analyzed and compared to control mice which received PBS. No significant differences in total- or trabecular BMD were observed between PBS and anti-IL1 or anti-TNF treated mice 10 days post i.a. injection with *S. aureus* Lpp (Figures 4C,D).

## Discussion

Tarkowski et al. demonstrated that hematogenous staphylococcal septic arthritis induces rapid systemic bone loss (Verdrengh et al., 2006). In the present study, we use a locally induced staphylococcal septic arthritis model to validate previous observations (Verdrengh et al., 2006), and further study the molecular and cellular mechanisms behind this effect. Our results demonstrate that local *S. aureus* septic arthritis causes systemic and local bone resorption. We found that the major players in bone resorption are the *S. aureus* Lpps that downregulate both total- and trabecular BMD. This effect is fully dependent on the lipid-moiety of Lpps. Interestingly, diacylated lipopeptides are more potent in inducing bone resorption than triacylated ones. Furthermore, monocyte depletion by i.v. injection of clodronate liposomes abrogates the osteoporotic effect of Lpps, suggesting that the recruited monocytes in local synovial tissue in septic arthritis may be the key mediators for systemic bone loss in septic arthritis.

The binding of RANKL, a member of the TNF family, to its receptor, RANK, results in the fusion, differentiation, activation, and survival of osteoclasts (Lacey et al., 1998; Liu and Zhang, 2015). It has been demonstrated in previous *ex vivo* studies that *S. aureus* stimulation induces bone resorption and osteoclast differentiation by increasing osteoblast. *Staphylococcus aureus* induced RANKL expression in osteoblasts was entirely controlled by TLR2, because osteoblasts isolated from TLR2-deficient mice does not respond to *S. aureus* stimulation (Kassem et al., 2016). It is not surprising that Lpps are capable to induce the bone resorption, as TLR2 is known to be the ligand to *S. aureus* lipoproteins and Lpps are one of the primary arthritogenic *S. aureus* components (Mohammad et al., 2019). We observed that the lipid moiety in Lpp must present to reduce the murine BMD. The protein alone shows no significant activity.

Interestingly, we have found that Lpl1(+sp) isolated from SA113 clone contained a mixture of di-acyl and tri-acyl lipid moiety structures at N-terminal. Indeed, *S. aureus* has been shown to produce both di-acylated Lpp (Tawaratsumida et al., 2009) and tri-acylated Lpp (Kurokawa et al., 2009) under environmental conditions (Kurokawa et al., 2012). Recently, Gardiner and colleagues have discovered two genes, namely N-acylation transferase system A (*lns*A) and B (*lns*B) which were involved in N-acylation of Lpp in *S. aureus* (Gardiner et al., 2020). However, under which environmental conditions *S. aureus* produces more di-acylated Lpp or tri-acylated Lpp particularly in infection is not known. Since we demonstrate that both synthetic lipopeptides Pam2Cys and Pam3Cys significantly reduce murine BMD, we further investigated whether such lipopeptides can be produced *in vivo*. We digested purified Lpl1 with trypsin and found that a mixture of lipid-CGKGNETK and lipid-CGK were produced with di- or tri-acylations. This indicates that lipopeptides similar to the synthetic Pam2Cys and Pam3Cys can also be produced in infection.

Previous studies have demonstrated that the lipopeptides Pam2Cys and Pam3Cys induce osteoclast differentiation and activation by upregulating RANKL expression and suppressing osteoprotegerin expression in osteoblasts (Kim et al., 2013). In the current study, we have demonstrated that Pam2Cys displays increased potency compared to Pam3Cys in bone resorptive capacities, which is in line with previous findings by Nguyen et al. (2017), as Pam2Cys is known to be more potent in activating immune response and causing acute inflammation than Pam3Cys.

Although *S. aureus* Lpps have demonstrated a potent arthritogenic effect, it is very likely that other components of the bacteria may contribute to the development of osteoporosis in septic arthritis. For example, repetitive inhalation of peptidoglycan has been shown to induce bone loss in mice (Dusad et al., 2013). Furthermore, lipopolysaccharides and peptidoglycan have been reported to have a synergistic effect on bone resorption and osteoclastogenesis (Kishimoto et al., 2012). We infer that Lpps and peptidoglycan may also exhibit similar synergistic effects, as seen by Lpps *in vitro* stimulation boosting peptidoglycan stimulation of NOD2 (Schaffler et al., 2014). Our data suggest that the effect of BMD loss induced by *S. aureus* Lpps is of a complex time-dependent nature. On the later time point when joint inflammation caused by Lpp was less pronounced, the difference between PBS and Lpps injected knees disappeared. In contrast, in the case of live bacterial septic arthritis, the differences between PBS and bacteria injected knees were apparent on day 7 and continued to grow until day 14 post infection. We speculate that the discrepancy in time kinetics of BMD loss between Lpps and live *S. aureus* was mainly due to the different courses of disease models. In the bacterial septic arthritis setting the joint inflammation persists and accelerates after infection, which leads to continuous decline of BMD. In Lpps-induced arthritis setting, the differences in BMD become less apparent on the later time points when the inflammation was resolved.

Of note, the difference was also found in total and trabecular BMD between PBS and healthy knees on day 14 post infection when mice were infected intraarticularly with live *S. aureus*. This strongly suggests that systemic inflammation induced by local knee infection has significant impact on the osteoclastogenesis. The mice with septic arthritis were mobile and had only minimal weight loss at the beginning of the disease. However, they may avoid using the infected leg due to pain or discomfort. Immobility is known to result in rapid bone loss (Chan, 2005). Avoiding load bearing due to pain in the infected leg might be one of the contributing factors to the bone loss.

The strong interaction between bone and immune cells contributes to the pathogenesis of osteoporosis in humans (Fischer and Haffner-Luntzer, 2021). It is known that monocytes are circulating osteoclast precursor cells, and that bone erosions in bone diseases, such as rheumatoid arthritis (RA), may be caused by the differentiation of classical monocytes into osteoclasts (Rana et al., 2018). Our previous data demonstrated that i.a injection of Lpps induces rapid infiltration of monocytes into the synovial tissue (Mohammad et al., 2019). Mature monocytes and macrophages can be transformed to osteoclasts when a suitable microenvironment is provided by bone marrow-derived stromal cells (Udagawa et al., 1990). Based on our findings, we hypothesize that recruitment of monocytes to infected joints and transformation of monocytes/macrophages to osteoclasts are the key steps of the rapid bone resorption in septic arthritis. However, further studies are warranted to elucidate detailed mechanisms in our animal models. Growth factors and cytokines such as macrophage colony-stimulating factor (M-CSF) and receptor activation of nuclear factor kappa-B ligand (RANKL) tightly regulate the activity and formation of osteoclasts. Both of these molecules are produced by synovial fibroblasts in the inflamed joints, and RANKL can also be produced by T-cells (Colucci et al., 2004) that are abundant in septic arthritis joints (Abdelnour et al., 1994). Moreover, differentiation of monocytes to osteoclasts can occur through stimulation with inflammatory cytokines such as TNF-α (Kim et al., 2005). Here, we also show that both Lpp and PBS injected mice with neutropenia exhibit lower BMD compared to control mice, which suggests that neutrophils exhibit a protective role in bone metabolism. In accordance with our results, it has been shown that patients with chronic neutropenia suffer from low BMD (Yakisan et al., 1997; Papadaki et al., 1999). In addition, the severity of the bone loss is strongly correlated with neutrophil counts (Papadaki et al., 1999). However, more studies are required to validate the results and further understand how the impact is mediated.

What is the clinical relevance of our findings? Advanced age and RA are risk factors for septic arthritis. Both RA and elderly patients are at a higher risk for developing osteoporosis. Our findings demonstrate that septic arthritis rapidly induces systemic BMD loss. To reduce the risk of bone fracture, we suggest examining all patients with a history of septic arthritis with bone densitometry, as osteoporosis is relatively easy to treat using bisphosphonate and RANKL inhibitors. Elderly and RA patients with a history of septic arthritis might have drastically increased risk for osteoporosis development and the anti-osteoporosis therapies could be introduced at the early stage of the disease. It has been demonstrated previously in a murine model that a combination of bisphosphonate, antibiotics, and anti-inflammatory treatment attenuates bone resorption in septic arthritis (Verdrengh et al., 2007), which suggests a new treatment modality for those patient groups.

## Data Availability Statement

The original contributions presented in the study are included in the article/Supplementary Material; further inquiries can be directed to the corresponding author. Furthermore, the mass spectrometry data have been deposited to Proteome X change via the MassIVE partner repository with the data-set identifier MassIVE MSV000088739 (https://massive.ucsd.edu/ProteoSAFe/static/massive.jsp). The data is provided as vendor specific .raw format and additionally as open .mzML format.

## Ethics Statement

The animal study was reviewed and approved by The Ethics Committee of Animal Research of Gothenburg approved all experiments conducted on mice. The mouse experiments were performed in accordance with the Swedish Board of Agriculture’s regulations and recommendations on animal experiments.

## Author Contributions

MS, MM, and TJ conceived the study. MS, MM, M-TN, ZH, AJ, CW, MF, and AA carried out the data collection and statistical analysis of the data. The manuscript was drafted by MS and TJ, and critically revised by MS, MM, M-TN, ZH, AJ, CW, MF, RP, AA, HH, FG, and TJ. All authors contributed to the article and approved the submitted version.

## Funding

This work was supported by the Swedish Medical Research Council (grant number 523-2013-2750 to TJ); grants from the Swedish state under the agreement between the Swedish Government and the county councils, the ALF-agreement (grant number ALFGBG-823941 to TJ, ALFGBG-770411 to AJ, and ALFGBG-926621 to RP); Professor Nanna Svartz Fond (grant number 2016-00117 to TJ and 2014-00058 and 2016-00154 to RP); the Swedish Rheumatism Association (grant numbers R-385441 and R-478421 to RP); the Swedish Medical Society (grant number SLS-505901 to RP); the Gothenburg Society of Medicine (grant number GLS-002/02 to MS and GLS-784641 to AJ); Stiftelsen Erik & Lily Philipson Minnesfond to MS; the Wilhelm and Martina Lundgren Foundation to (TJ, AA, AJ, and RP); Rune och Ulla Amlövs Stiftelse för Neurologisk och Reumatologisk Forskning (grant number 2016-075 to TJ and 2015-00053 and 2017-129 to AJ); Adlerbertska Forskningsstiftelsen to (MM, AA, AJ, and TJ); Inger Bendix stiftelse to (AJ); Sahlgrenska University Foundations to (AJ); Kungl. Vetenskapsakademiens stiftelser (grant number ME2015-0119 to AA); E och K.G. Lennanders stipendiestiftelse to (MM and AA); Magnus Bergvalls Stiftelse (grant numbers 2017-01958 and 2018-02797 to AA); and Institute of Medicine, Gothenburg University. FG was funded by the Deutsche Forschungsgemeinschaft (DFG) Germany’s Excellence Strategy—EXC 2124—390838134 “Controlling Microbes to Fight Infections.” HH acknowledges financial support by the Deutsche Forschungsgemeinschaft (DFG; INST 211/802-1 FUGG). The funders had no role in study design, data collection and analysis, decision to publish, or preparation of the manuscript.

## Conflict of Interest

The authors declare that the research was conducted in the absence of any commercial or financial relationships that could be construed as a potential conflict of interest.

## Publisher’s Note

All claims expressed in this article are solely those of the authors and do not necessarily represent those of their affiliated organizations, or those of the publisher, the editors and the reviewers. Any product that may be evaluated in this article, or claim that may be made by its manufacturer, is not guaranteed or endorsed by the publisher.

## Abbreviations

BMD: Bone mineral density
i.a.: Intra-articularly
Lpps: Bacterial lipoproteins
Lpl1(+sp): Purified *S. aureus* specific model lipoprotein carrying the lipid moity
Lpl1(−sp): Lpl1 protein lacking the lipid moiety
Pam2Cys: (Pam_2_CSK_4_) synthetic dipalmitoylated lipopeptide that mimics the acylated amino terminus of bacterial lipoproteins
Pam3Cys: (Pam_3_CSK_4_) synthetic tripalmitoylated lipopeptide
RA: Rheumatoid arthritis
TLRs: Toll-like receptor(s)
