# Supplementary material for: Lipoproteins Cause Bone Resorption in a Mouse Model of Staphylococcus aureus Septic Arthritis
Source: Front Microbiol. 2022 Mar 9;13:843799. doi: 10.3389/fmicb.2022.843799 (PMC8959583; doi:10.3389/fmicb.2022.843799)
Supplement: Supplementary file 1 [file Data_Sheet_1.PDF]

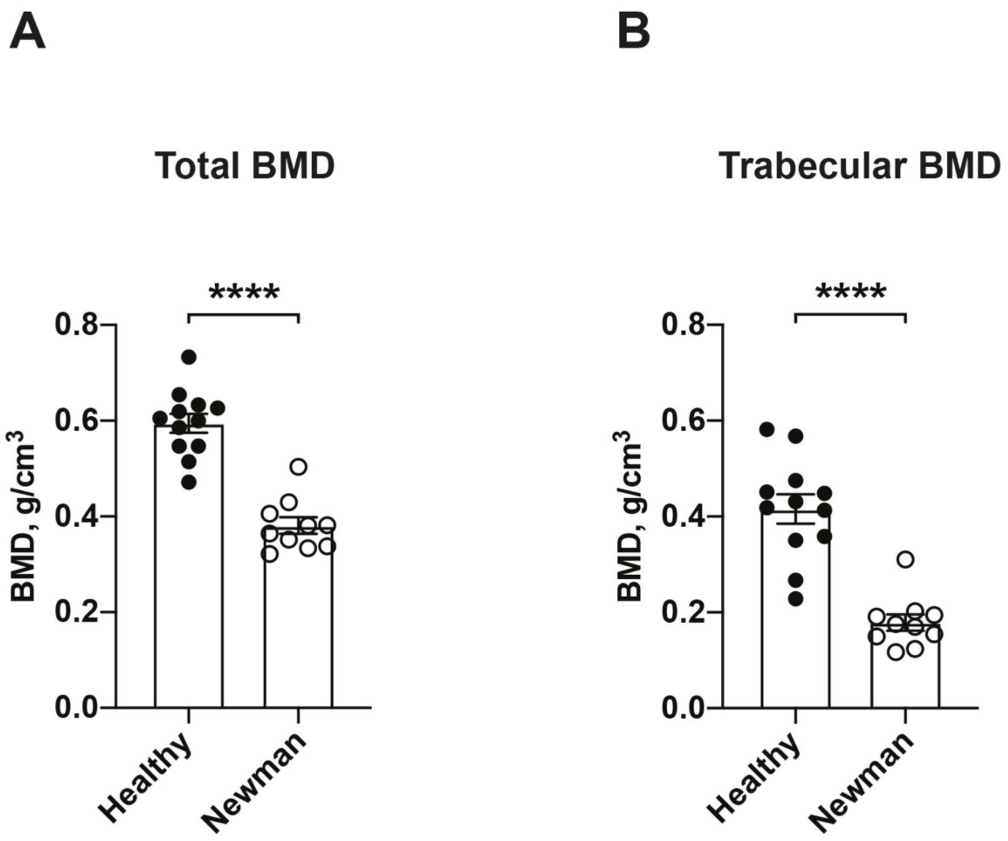

Supplementary Figure 1. Intra-articular injection of *Staphylococcus aureus* Newman strain induces bone resorption. The total- (**A**) and trabecular (**B**) bone mineral density (BMD, g/cm^3) were measured in NMRI mice 10 days after intra-articular (i.a.) injection with 20µl of *Staphylococcus aureus* (*S. aureus*) Newman strain (4x10^3 colony-forming units [CFU]/knee) (n=10 knee joints) compared with healthy NMRI mice (n=12 knee joints) i.a. injected with 20µl of PBS. Statistical evaluations were performed using the Mann-Whitney *U* test with data expressed as the mean ± standard error of the mean (*****P* <0.0001).

Identification of lipopeptides by LC-MS

After tryptic digestion of the purified protein, the resulting lipopeptides were subjected to LC-MS analysis. Two different types of tryptic peptide parts could be detected, one directly cleaved after the first lysine (lipid-CGK), the other one by cleavage after the second lysine (lipid-CGKGNETK), i.e. with one missed cleavage side. The detected short lipopeptides are mostly singly charged, the more abundant CGKGNETK-containing lipopeptides can be detected as singly and doubly charged molecules, with the doubly charged ones being the more intensive.

In the following figures, the extracted ion chromatograms (EICs) of lipopeptides identified in the protein sample are displayed (**Supplementary Figure S2-S4**). In **Supplementary Figure 5 and 6**, the MS/MS of the respective *m/z* are displayed. An exemplary MS/MS spectrum of the triacyl 48:0 lipopeptide (*m/z* 813.0610) with the corresponding structure and fragmentation is depicted in **Supplementary Figure 7**.

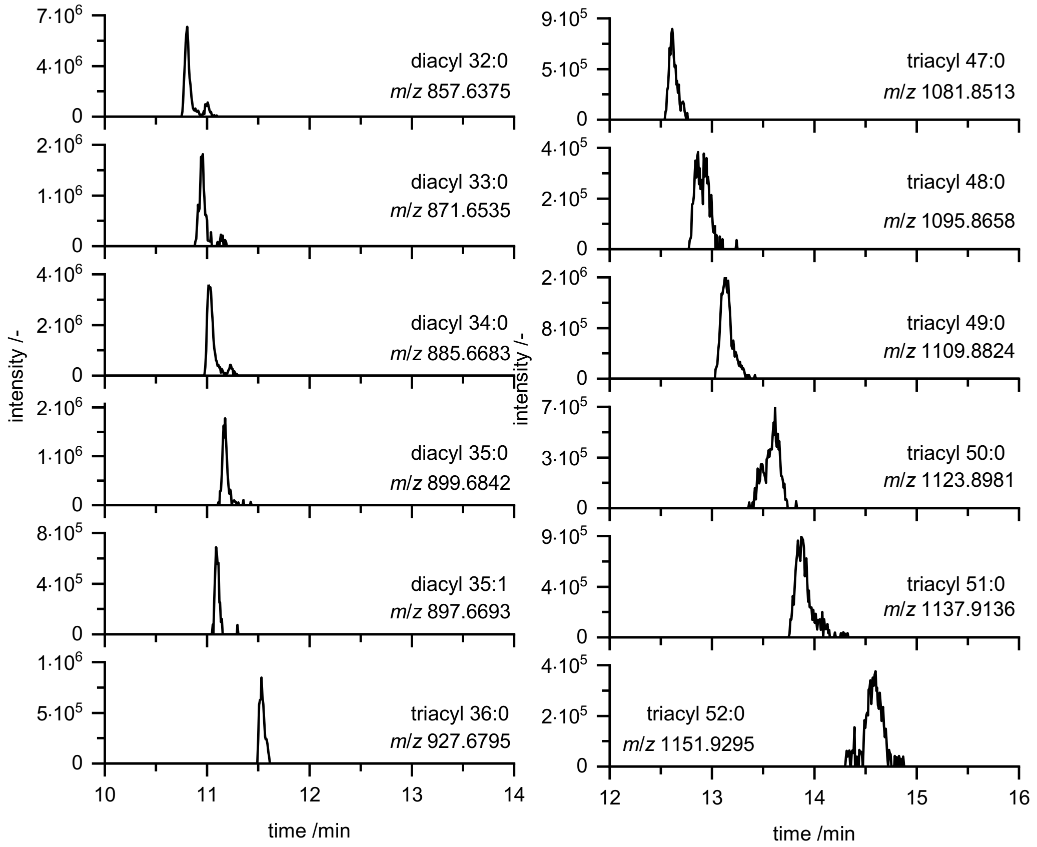

Supplementary Figure S2. EICs of lipopeptides containing the peptide moiety CGK.

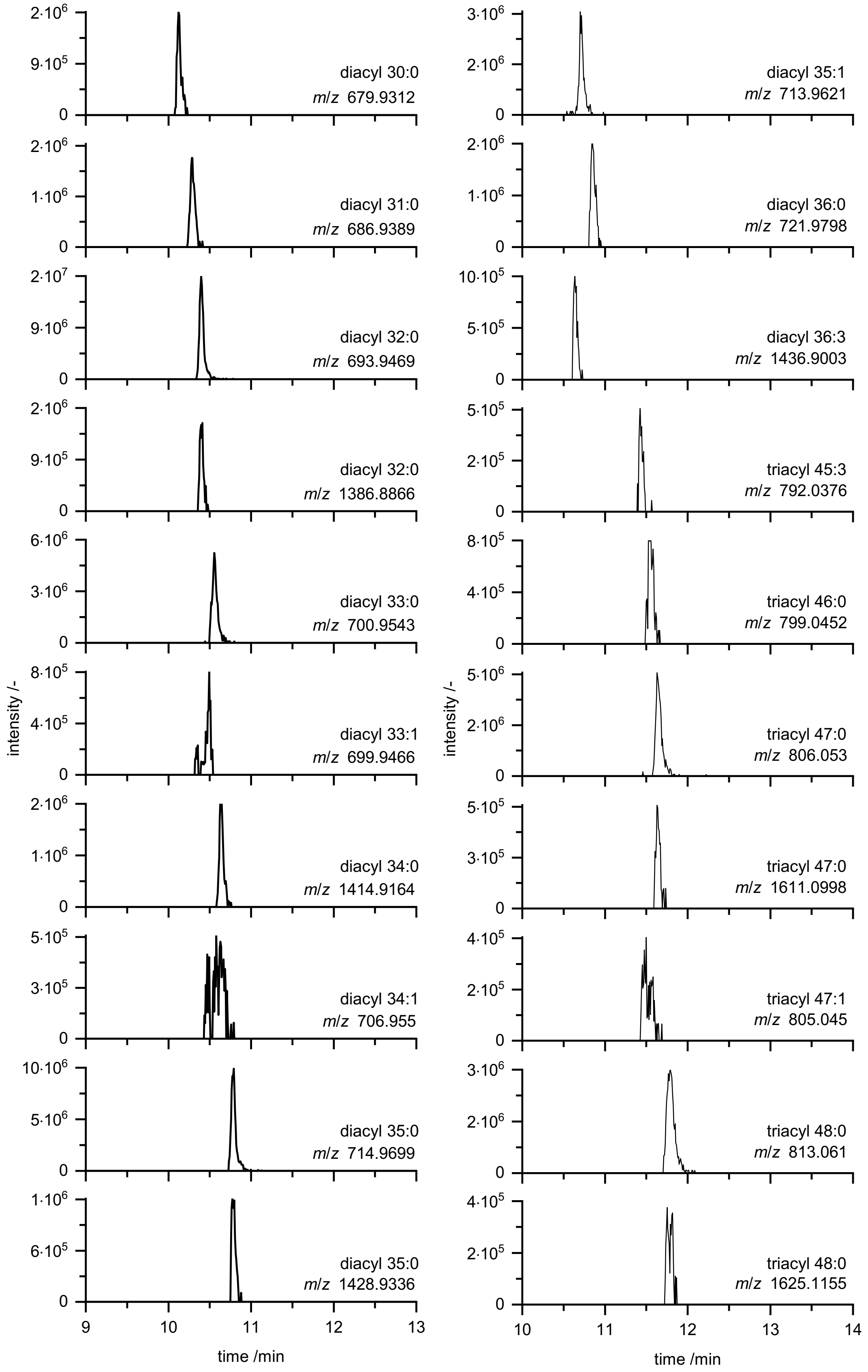

Supplementary Figure S3. EICs of lipopeptides containing the peptide moiety CGKGNETK.

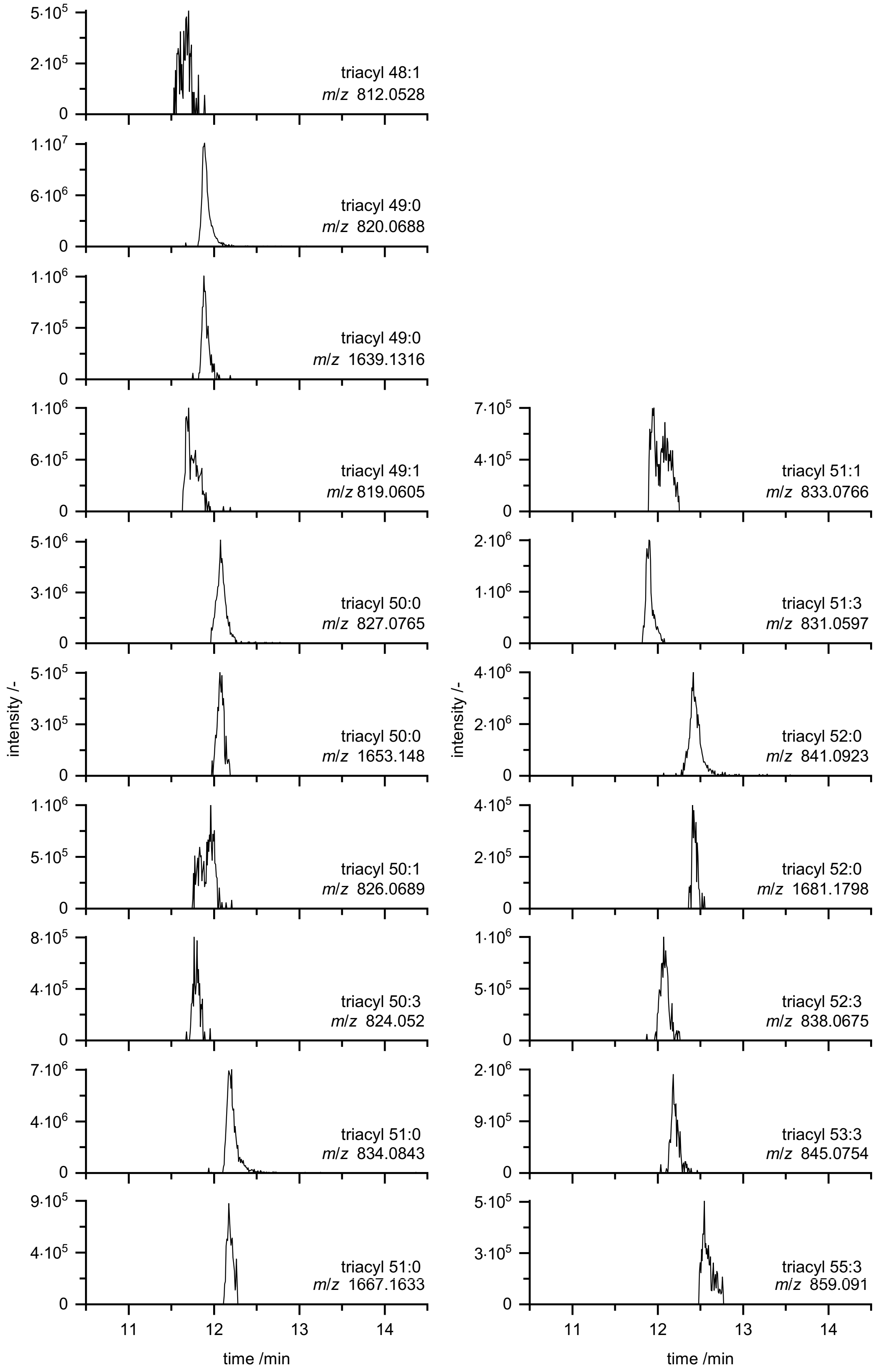

Supplementary Figure S4. EICs of lipopeptides containing the peptide moiety CGKGNETK – continued.

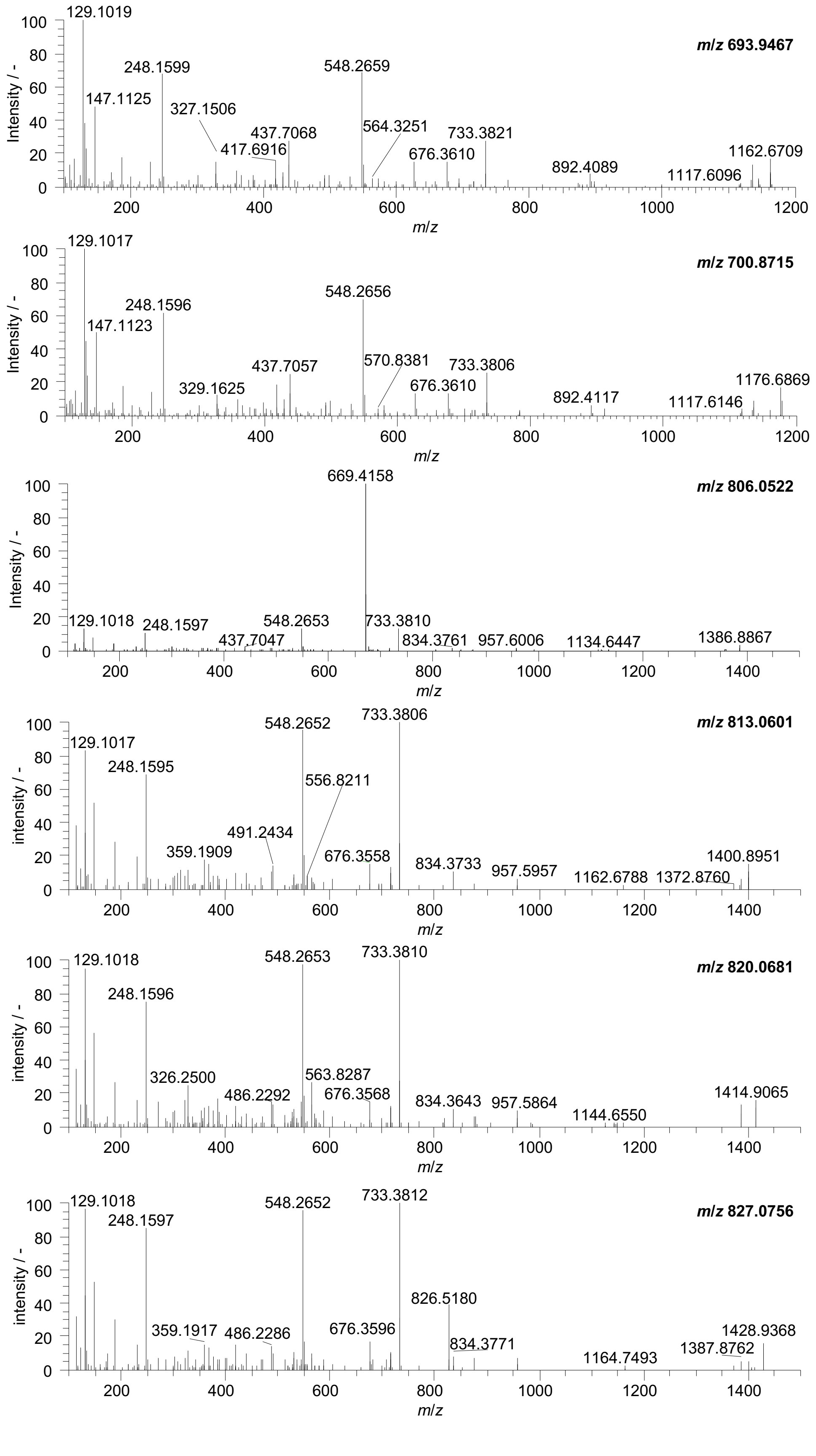

Supplementary Figure S5. MS/MS fragmentation spectra obtaining by isolating the displayed *m/z* and applying a collision energy of 30 eV.

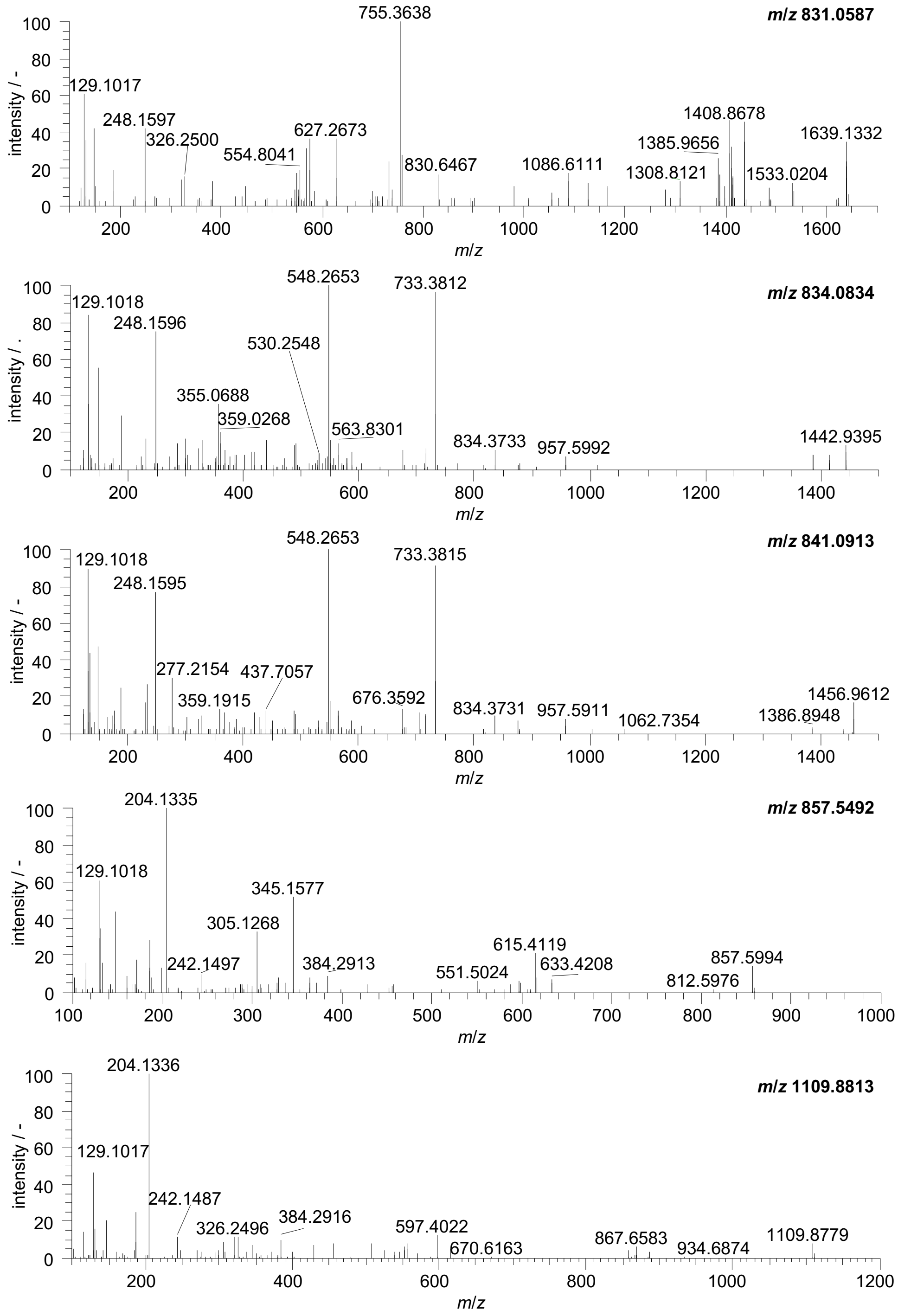

Supplementary Figure S6: MS/MS fragmentation spectra obtaining by isolating the displayed *m*/*z* and applying a collision energy of 30 eV – continued.

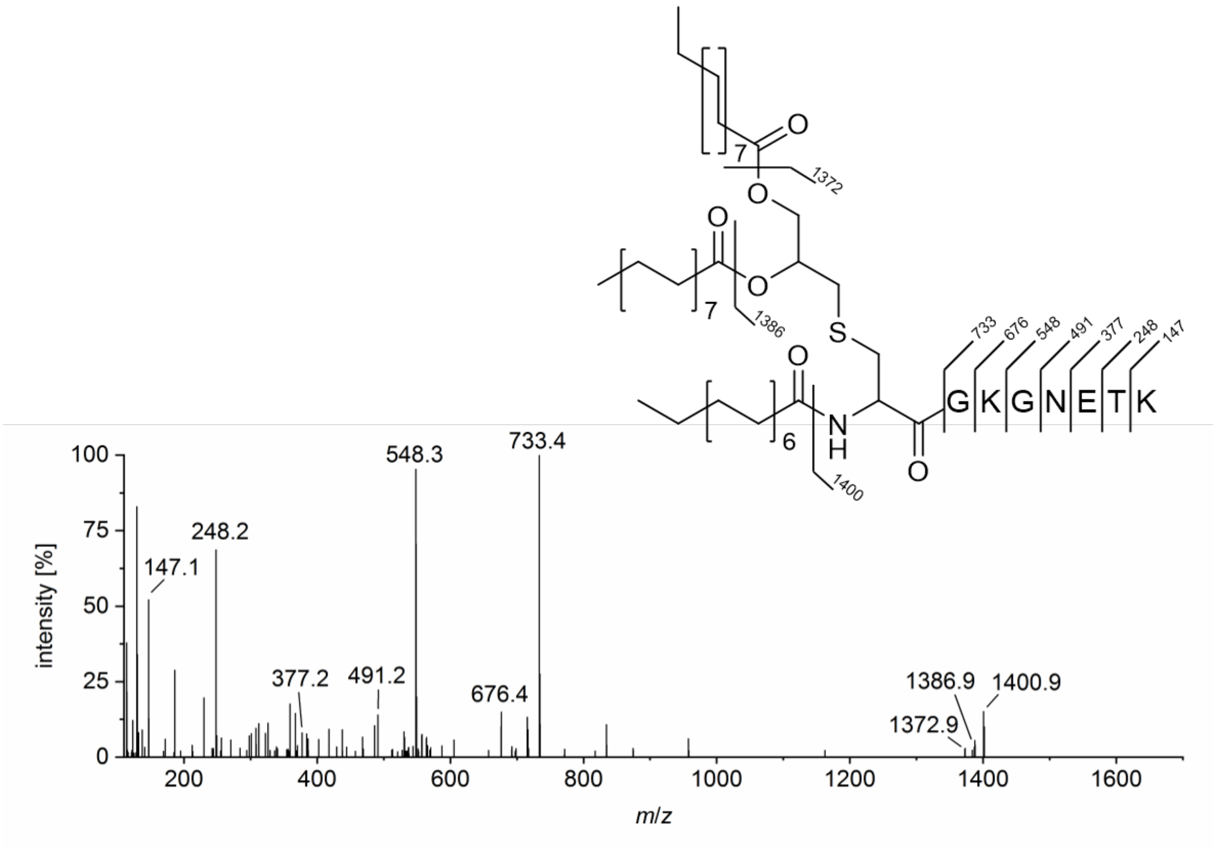

Supplementary Figure S7. Product ion spectrum (collision energy of 30 eV) of the triacyl 48:0 lipopeptide at *m/z* 813.0610 including a schematic structure and the corresponding fragmentation. One digit is displayed for better visualization. Note that the exact position of the acyl chains can´t be determined with the applied method and the shown positions are a suggestion.

The following **Supplementary Table S1** lists the lipopeptides identified using HRMS, retention time and the fragmentation spectra. In addition, lipopeptides are listed, that are putatively identified using HRMS and the retention time. The designation diacyl in the table corresponds to two fatty acids, which could either be a lyso-lipopeptide or a diacyl-lipopeptide.

Supplementary Table Error! No text of specified style in document.**.** Lipopeptides putatively identified using by LC-MS(/MS). Displayed are the lipid and peptide moieties of the tryptic lipopeptides, the theoretical and measured *m*/*z* and deviation in ppm, the ion, retention time (t_R) and the area of the peak in the extracted ion chromatogram. Marked with an asterisk (*) are species, where a fragmentation spectrum is available.

Lipid moiety	Peptide	*m/z*	*m/z*$_{theor}$	ppm	Ion	t_R	Area
diacyl 32:0*	CGK	857.6375	857.6396	2.5	$[M+H]^+$	10.8	$2.0 \cdot 10^7$
diacyl 33:0	CGK	871.6535	871.6552	1.9	$[M+H]^+$	11.0	$6.2 \cdot 10^6$
diacyl 34:0	CGK	885.6683	885.6709	2.9	$[M+H]^+$	11.0	$1.4 \cdot 10^7$
diacyl 35:0	CGK	899.6842	899.6865	2.6	$[M+H]^+$	11.2	$6.0 \cdot 10^6$
diacyl 35:1	CGK	897.6693	897.6709	1.7	$[M+H]^+$	11.1	$1.8 \cdot 10^6$
triacyl 36:0	CGK	927.6795	927.6814	2.1	$[M+H]^+$	11.5	$2.7 \cdot 10^6$
triacyl 47:0	CGK	1081.8513	1081.8536	2.1	$[M+H]^+$	12.7	$4.0 \cdot 10^6$
triacyl 48:0	CGK	1095.8658	1095.8692	3.1	$[M+H]^+$	13.0	$3.6 \cdot 10^6$
triacyl 49:0*	CGK	1109.8824	1109.8849	2.2	$[M+H]^+$	13.2	$1.0 \cdot 10^7$
triacyl 50:0	CGK	1123.8981	1123.9005	2.1	$[M+H]^+$	13.7	$5.2 \cdot 10^6$
triacyl 51:0	CGK	1137.9136	1137.9162	2.3	$[M+H]^+$	14.0	$7.2 \cdot 10^6$
triacyl 52:0	CGK	1151.9295	1151.9318	2.0	$[M+H]^+$	14.8	$3.2 \cdot 10^6$
diacyl 30:0	CGKGNETK	679.9312	679.9320	1.2	$[M+2H]^{2+}$	10.1	$5.6 \cdot 10^6$
diacyl 31:0	CGKGNETK	686.9389	686.9399	1.5	$[M+2H]^{2+}$	10.3	$7.3 \cdot 10^6$
diacyl 32:0*	CGKGNETK	693.9469	693.9477	1.2	$[M+2H]^{2+}$	10.4	$5.9 \cdot 10^7$
diacyl 32:0	CGKGNETK	1386.8866	1386.8892	1.9	$[M+H]^+$	10.4	$4.9 \cdot 10^6$
diacyl 33:0*	CGKGNETK	700.9543	700.9555	1.8	$[M+2H]^{2+}$	10.6	$2.0 \cdot 10^7$
diacyl 33:1	CGKGNETK	699.9466	699.9477	1.6	$[M+2H]^{2+}$	10.5	$2.2 \cdot 10^6$
diacyl 34:0	CGKGNETK	1414.9164	1414.9205	2.9	$[M+H]^+$	10.6	$8.3 \cdot 10^6$
diacyl 34:1	CGKGNETK	706.9550	706.9555	0.6	$[M+2H]^{2+}$	10.6	$2.0 \cdot 10^6$
diacyl 35:0*	CGKGNETK	714.9699	714.9712	1.8	$[M+2H]^{2+}$	10.8	$3.4 \cdot 10^7$
diacyl 35:0	CGKGNETK	1428.9336	1428.9361	1.8	$[M+H]^+$	10.8	$3.9 \cdot 10^6$
diacyl 35:1*	CGKGNETK	713.9621	713.9633	1.8	$[M+2H]^{2+}$	10.7	$1.1 \cdot 10^7$
diacyl 36:0	CGKGNETK	721.9798	721.9790	1.2	$[M+2H]^{2+}$	10.9	$8.0 \cdot 10^6$
diacyl 36:3	CGKGNETK	1436.9003	1436.9048	3.2	$[M+H]^+$	10.6	$3.2 \cdot 10^6$
triacyl 45:3	CGKGNETK	792.0376	792.0390	1.8	$[M+2H]^{2+}$	11.4	$1.4 \cdot 10^6$
triacyl 46:0	CGKGNETK	799.0452	799.0469	2.0	$[M+2H]^{2+}$	11.6	$3.8 \cdot 10^6$
triacyl 47:0*	CGKGNETK	806.0530	806.0547	2.1	$[M+2H]^{2+}$	11.7	$2.0 \cdot 10^7$
triacyl 47:0	CGKGNETK	1611.0998	1611.1032	2.1	$[M+H]^+$	11.7	$1.7 \cdot 10^6$
triacyl 47:1	CGKGNETK	805.0450	805.0469	2.3	$[M+2H]^{2+}$	11.5	$2.3 \cdot 10^6$
triacyl 48:0*	CGKGNETK	813.0610	813.0625	1.9	$[M+2H]^{2+}$	11.8	$2.0 \cdot 10^7$
triacyl 48:0	CGKGNETK	1625.1155	1625.1188	2.0	$[M+H]^+$	11.8	$1.5 \cdot 10^6$
triacyl 48:1	CGKGNETK	812.0528	812.0547	2.3	$[M+2H]^{2+}$	11.7	$2.9 \cdot 10^6$
triacyl 49:0*	CGKGNETK	820.0688	820.0703	1.8	$[M+2H]^{2+}$	11.9	$5.9 \cdot 10^7$

triacyl 49:0	CGKGNETK	1639.1316	1639.1345	1.7	$[M+H]^{+}$	11.9	$5.6 \cdot 10^{6}$
triacyl 49:1	CGKGNETK	819.0605	819.0625	2.5	$[M+2H]^{2+}$	11.7	$7.6 \cdot 10^{6}$
triacyl 50:0*	CGKGNETK	827.0765	827.0782	2.1	$[M+2H]^{2+}$	12.1	$3.1 \cdot 10^{7}$
triacyl 50:0	CGKGNETK	1653.1480	1653.1501	1.3	$[M+H]^{+}$	12.1	$2.6 \cdot 10^{6}$
triacyl 50:1	CGKGNETK	826.0689	826.0703	1.7	$[M+2H]^{2+}$	12.0	$7.9 \cdot 10^{6}$
triacyl 50:3	CGKGNETK	824.0520	824.0547	3.3	$[M+2H]^{2+}$	11.8	$3.3 \cdot 10^{6}$
triacyl 51:0*	CGKGNETK	834.0843	834.0860	2.0	$[M+2H]^{2+}$	12.2	$4.6 \cdot 10^{7}$
triacyl 51:0	CGKGNETK	1667.1633	1667.1658	1.5	$[M+H]^{+}$	12.2	$3.8 \cdot 10^{6}$
triacyl 51:1	CGKGNETK	833.0766	833.0782	1.9	$[M+2H]^{2+}$	12.0	$7.3 \cdot 10^{6}$
triacyl 51:3*	CGKGNETK	831.0597	831.0625	3.3	$[M+2H]^{2+}$	11.9	$1.0 \cdot 10^{7}$
triacyl 52:0*	CGKGNETK	841.0923	841.0938	1.8	$[M+2H]^{2+}$	12.5	$2.6 \cdot 10^{7}$
triacyl 52:0	CGKGNETK	1681.1798	1681.1814	1.0	$[M+H]^{+}$	12.5	$1.4 \cdot 10^{6}$
triacyl 52:3	CGKGNETK	838.0675	838.0703	3.3	$[M+2H]^{2+}$	12.1	$5.5 \cdot 10^{6}$
triacyl 53:3	CGKGNETK	845.0754	845.0782	3.3	$[M+2H]^{2+}$	12.2	$7.5 \cdot 10^{6}$
triacyl 55:3	CGKGNETK	859.0910	859.0938	3.2	$[M+2H]^{2+}$	12.6	$3.1 \cdot 10^{6}$
